# Accuracy and feasibility of a novel fine hand motor skill assessment using computer vision object tracking

**DOI:** 10.1038/s41598-023-29091-0

**Published:** 2023-02-01

**Authors:** Bokkyu Kim, Christopher Neville

**Affiliations:** Department of Physical Therapy Education, College of Health Professions, SUNY Upstate Medical University, Syracuse, NY USA

**Keywords:** Outcomes research, Motor control, Rehabilitation

## Abstract

We developed a computer vision-based three-dimension (3D) motion capture system employing two action cameras to examine fine hand motor skill by tracking an object manipulated by a hand. This study aimed to examine the accuracy and feasibility of this approach for detecting changes in a fine hand motor skill. We conducted three distinct experiments to assess the system's accuracy and feasibility. We employed two high-resolution, high-frame-rate action cameras. We evaluated the accuracy of our system in calculating the 3D locations of moving object in various directions. We also examined the system's feasibility in identifying improvement in fine hand motor skill after practice in eleven non-disabled young adults. We utilized color-based object detection and tracking to estimate the object's 3D location, and then we computed the object's kinematics, representing the endpoint goal-directed arm reaching movement. Compared to ground truth measurements, the findings demonstrated that our system can adequately estimate the 3D locations of a moving object. We also showed that the system can be used to measure the endpoint kinematics of goal-directed arm reaching movements to detect changes in fine hand motor skill after practice. Future research is needed to confirm the system's reliability and validity in assessing fine hand motor skills in patient populations.

## Introduction

Kinematic examination of human movement is critical in rehabilitation research and clinical practice to assess motor function in people with movement system issues caused by neurologic or musculoskeletal disorders. Kinematic measures, in particular, are valuable for objectively measuring changes in motor task performance in both non-disabled individuals and patients with motor deficits^1^. In the past, kinematic analysis of human movement was only possible in research laboratories using expensive motion capture devices such as marker-based optical motion capture cameras. Rapid advancements in computer vision and machine learning/deep learning have recently made human movement kinematic analysis more accessible in clinical settings^2–6^. We can now record movement kinematics in an outdoor sports field for soccer players^6^ or under the water in a swimming pool for professional swimmers^7^ using motion sensors, such as inertial measurement units (IMU) sensors or markerless motion capture systems based on color cameras.

The benefits of the markerless motion capture system over the marker-based motion capture system include removing differences in marker placement to minimize inter-session measurement variability, minimizing patient/subject preparation time, and cost-effectiveness^7–18^. Previous studies on markerless motion capture systems concentrated on gait or lower extremity movements^11,13,15,19^. Several recent studies have also proposed a computer vision-based markerless motion capture approach for measuring upper extremity and hand motor function in people with neurologic disorders^20–23^. These markerless motion capture systems primarily utilized 3-dimensional (3D) depth-sensing cameras, such as the Kinect, to estimate joint kinematics. Researchers demonstrated a high degree of accuracy, validity, and reliability for various markerless motion capture systems to record human body kinematics by using a gold-standard marker-based motion capture system^11,13,14,19,24,25^.

We developed a new computer vision-based markerless motion capture system that can recognize and track objects that a person manipulates in addition to recording human joint kinematics. Using this method, we may also examine improvement in motor performance or acquisition of fine hand motor skills, especially relevant to object manipulation. However, the efficacy of this technique in testing human upper extremity motor abilities by detecting and tracking an item manipulated by a person has not been properly evaluated.

The purpose of this research is to test the accuracy of a newly developed object tracking motion capture system for measuring the kinematics of an object manipulated to quantify a fine hand motor skill. We developed a computer vision-based markerless motion capture system using two commercially available action cameras. This system is capable of tracking an object that represents the endpoint of upper extremity (UE) motor performance. We carried out two experiments to select optimal video recording parameters, and to evaluate the system's accuracy in tracking an object and estimating its 3-dimensional position at various recording settings in simulated circumstances. Furthermore, we conducted a third pilot experiment with non-disabled young adults to apply the same technique in a new setting to determine the if the system can detect changes in fine hand motor skill that may indicate learning gains.

In this study, we describe all three experiments to show how we identified the optimal video recording parameters and settings for the stereo camera system from Experiments 1 and 2, which are used in Experiment 3.

## Methods

### Experiment 1: Validation of the stereo camera system accuracy in estimating the object positions during sagittal plane (anterior–posterior) movement

This experiment was carried out (1) to determine the optimal baseline distance for the stereo camera setup and (2) to assess the system's accuracy in estimating the distance between the camera and the object. The object is manually moved from the camera to 1-m distance from the camera in an anterior–posterior direction. Two cameras were used to record the object's movement. The object was then detected and tracked with the use of a custom programming script, and the kinematics of the object movement were evaluated to determine the distance between the camera and the object.

#### Experimental setup

**Figure 1:**
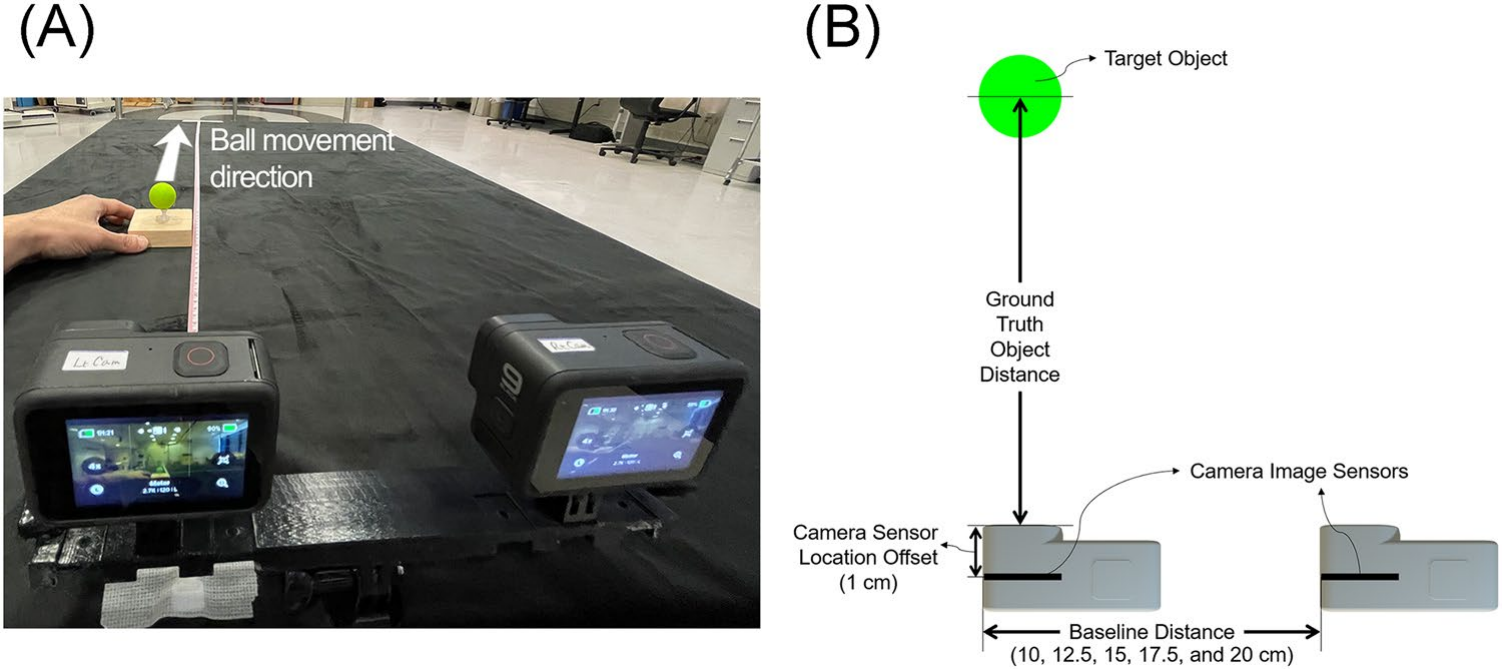
Experiment 1 setup. (**A**) A picture of the experiment's workspace. The table was draped with black cloth. A tape measure was placed on the middle of the table. Each 0.1 m was marked from 0.3 to 1 m. The stereo camera system was placed on the table. The target object is 0.3 m away in front of the left camera. The object was then manually moved in the z (anterior–posterior) direction at 0.1 m increments to a distance of 1 m from the camera. The video was taken at a frame rate of 24 frames per second (fps). (**B**) The experiment workspace from superior view. Two cameras were set apart at each baseline distance for each trial. In the data analysis, the camera image sensor offset (1 cm) was added to the estimated distance between the camera and the object.

Two high-resolution, high-frame-rate action cameras (GoPro Hero 9, San Mateo, CA, USA) were mounted to a dual twin mount adaptor with various baseline distances (10, 12.5, 15, 17.5, and 20 cm—see Fig. 1). A green ball was put on a table at 0.3 m away from the left camera of the stereo camera system (Fig. 1). The dual twin mount adapter was created using a 3D printer. The camera capture resolution was set to 2.7 K (2704 × 1520 pixels) and the recording rate was set at 24 frames per second (fps). Supplementary Table 2 summarizes more detailed video capture setup information.

#### Stereo camera calibration

The stereo camera system was calibrated using a checkerboard pattern. The checkerboard pattern was printed in black and white and then glued on a hardboard. An investigator positioned the checkerboard in front of the camera system at distances ranging from 0.3 to 1 m, while the stereo camera system captured checkerboard pictures at 120 frames per second. For the best calibration accuracy, checkerboard pattern pictures were acquired at a distance comparable to the distance between the camera and the object. Furthermore, the angle between the checkerboard and the camera plane remained less than 45°.

The stereo camera calibration was performed using the MATLAB (version R2020a, MathWorks, Inc., Natick, MA, USA) Stereo Camera Calibration Toolbox. Calibration videos were imported into the MATLAB workspace, and videos were synced by utilizing a custom MATLAB script to cross-correlate audio data between two video files. Each video data was then exported to a JPEG picture file every 50 frames. This procedure generates approximately 100 calibration images for each camera. The stereo camera calibration was performed using these extracted calibration images. Then, the calibration was performed again without image pairs with reprojection errors greater than 0.6 pixels. (Supplementary Fig. 2) This approach results in a more accurate calibration parameter with an average reprojection error of less than 0.5 pixels.

#### Data acquisition

We utilized an audio file to trigger the simultaneous video recording of two cameras. We used a Bluetooth speaker to play the audio file. The audio file plays an audio command—"GoPro start recording"—followed by a 1-s whistle sound. With the audio command, the action cameras started recording automatically. A green ball attached to a woodblock was put 0.3 m away from the left camera (Fig. 4). When the video capture began, the green ball travelled from the 0.3-m position to the 1-m point along with the anterior–posterior axis, increasing the distance with each 0.1-m step. Every 0.1-m step, the ball came to a complete stop for a second. The video capturing was stopped using a remote controller after the ball was at a 1-m distance from the camera. The data collection procedure was repeated with various camera baseline distances. Five different baseline distances (10, 12.5, 15, 17.5, and 20 cm) were used in this experiment.

#### Data analysis

Video data were imported to a laptop PC and pre-processed using a custom MATLAB script. Video pre-processing included video synchronization and un-distortion. Video synchronization was performed using cross-correlation between audio data from the video files. We established the lag index with the highest cross-correlation coefficient using ‘xcorr’ function. Video data un-distortion was conducted using the ‘stereocamera calibration parameters’ from the calibration process.

After pre-processing, each frame was converted from Red, Green, and Blue (RGB) to Hue, Saturation, and Value (HSV) color space. To improve the efficiency of the imaging process, the image frame was cropped to a target area to search for the target object (i.e., the green ball). Then the image was filtered with a pre-defined HSV color range and binarized. Then a binary mask was generated for pixels where all hue, saturation, and value binary data are 1. Then all those pixels less than 100 px were filtered out and filled holes in the binary mask using the 'imfill' function. Lastly, a morphological close operation was performed on the binary mask image using the 'strel' and 'imclose' functions.

**Figure 2:**
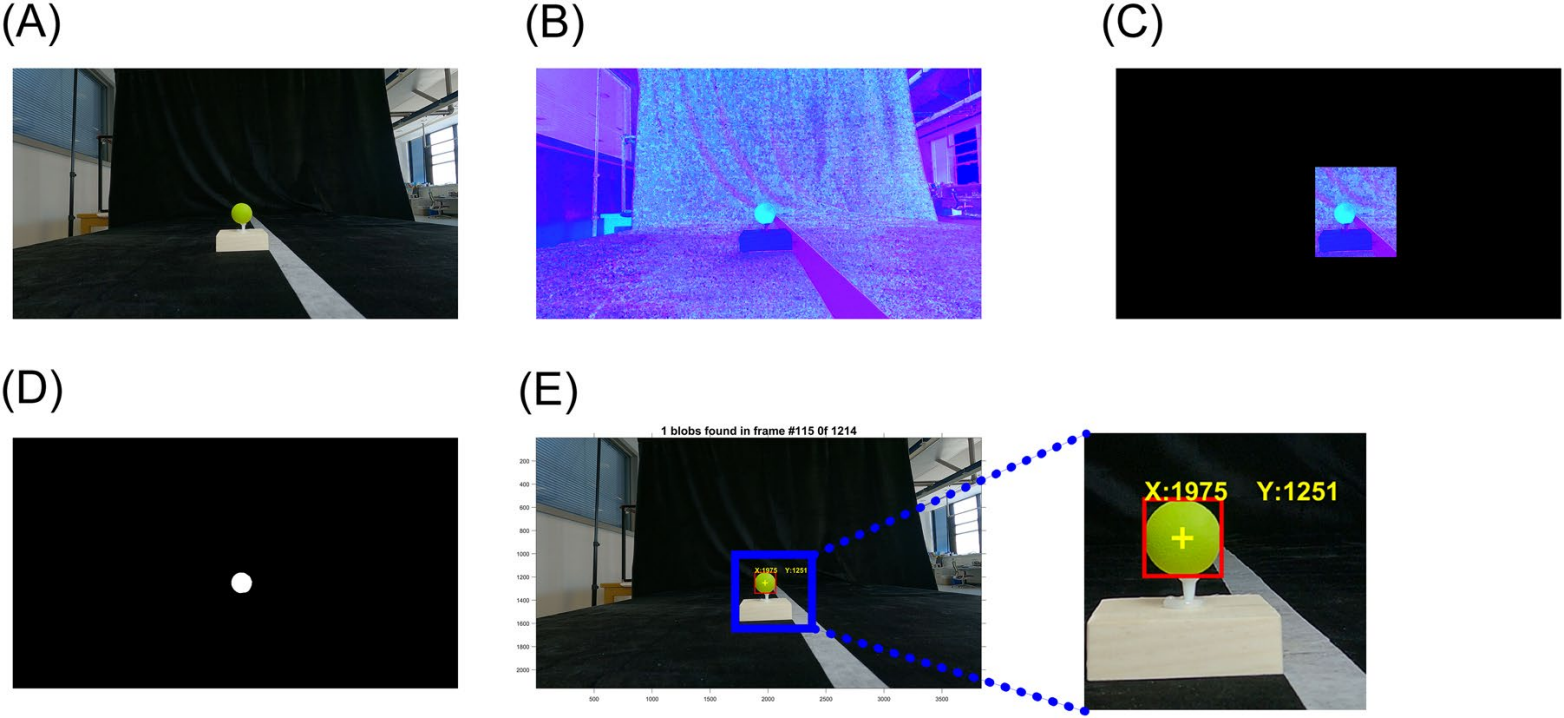
Experiment 1 object detection process. (**A**) Raw image in RGB color space. (**B**) Image in HSV color space. (**C**) Cropped HSV image. (**D**) Binary mask of the target object. (**E**) The final result of the object detection with bounding box (red square) and centroid (yellow cross). The Cartesian coordinate of the centroid is displayed in yellow text.

Using the binary mask image from the above step, we performed a blob analysis to bound the target object in a rectangular box. Then the centroid of the bound box was calculated on the x- and y-axis in pixel (Fig. 2). The green object was detected across all frames of the video data from two cameras. The green ball's three-dimensional (3-D) centroid positions were calculated using the 'triangulate' function with centroid data from two cameras. Then the resultants of the 3-D position data were calculated to estimate the distance between the camera and the green ball. The resultant data were filtered using a 3rd order Butterworth filter with a cutoff frequency of 3 Hz.

Tangential velocity was calculated as the first derivative of the filtered resultant. Movement onset and movement offset were automatically labelled using the velocity profile. Movement onset was defined as the first frame of the ball movement, where the tangential velocity is above 10% of the peak velocity^2^. Movement offset was defined as the last frame of the ball movement, where the tangential velocity is above 10% of the peak velocity^2^. (see Supplementary Fig. 6) An investigator (BK) visually inspected the individual trial movement onset and offset to determine if these were correctly labelled. Then we identified when the ball was not moving and calculated the distance between the ball and the left camera when the ball was not moving. A 1-cm was added to the estimated distance, given that the origin of the 3-D coordinate was the center of the left camera sensor, while the physical ground truth distance between the camera and the object was calculated from the camera lens. (Fig. 1B) The distance estimation error was calculated by subtracting the ground truth distance from the estimated distance from the video data.

Kruskal–Wallis test was performed to examine the difference in the distance estimation error between different baseline settings. A separate Kruskal–Wallis test was also conducted to examine the difference in the estimation error between different object distances from the camera.

### Experiment 2: Validation of the stereo camera system accuracy on the estimation of object positions during coronal plane movement

This experiment was carried out to assess the accuracy of the stereo camera system in estimating the position of a fast-moving object, such as a pendulum. We used a pendulum simulation, which was displayed onto a high-refresh-rate laptop PC monitor. The pendulum position was estimated incorrectly in our preliminary experiment, which used a passive marker-based 3-D optical motion capture system. As a result, rather than employing the gold-standard motion capture equipment, we used a pendulum simulation presented on a monitor with a high refresh rate of 300 Hz. This method allows us to compare pendulum kinematics estimated from our system to simulated pendulum kinematic data, which provides more accurate kinematic measurements of pendulum movement than the optical motion capture system.

#### Setup

**Figure 3:**
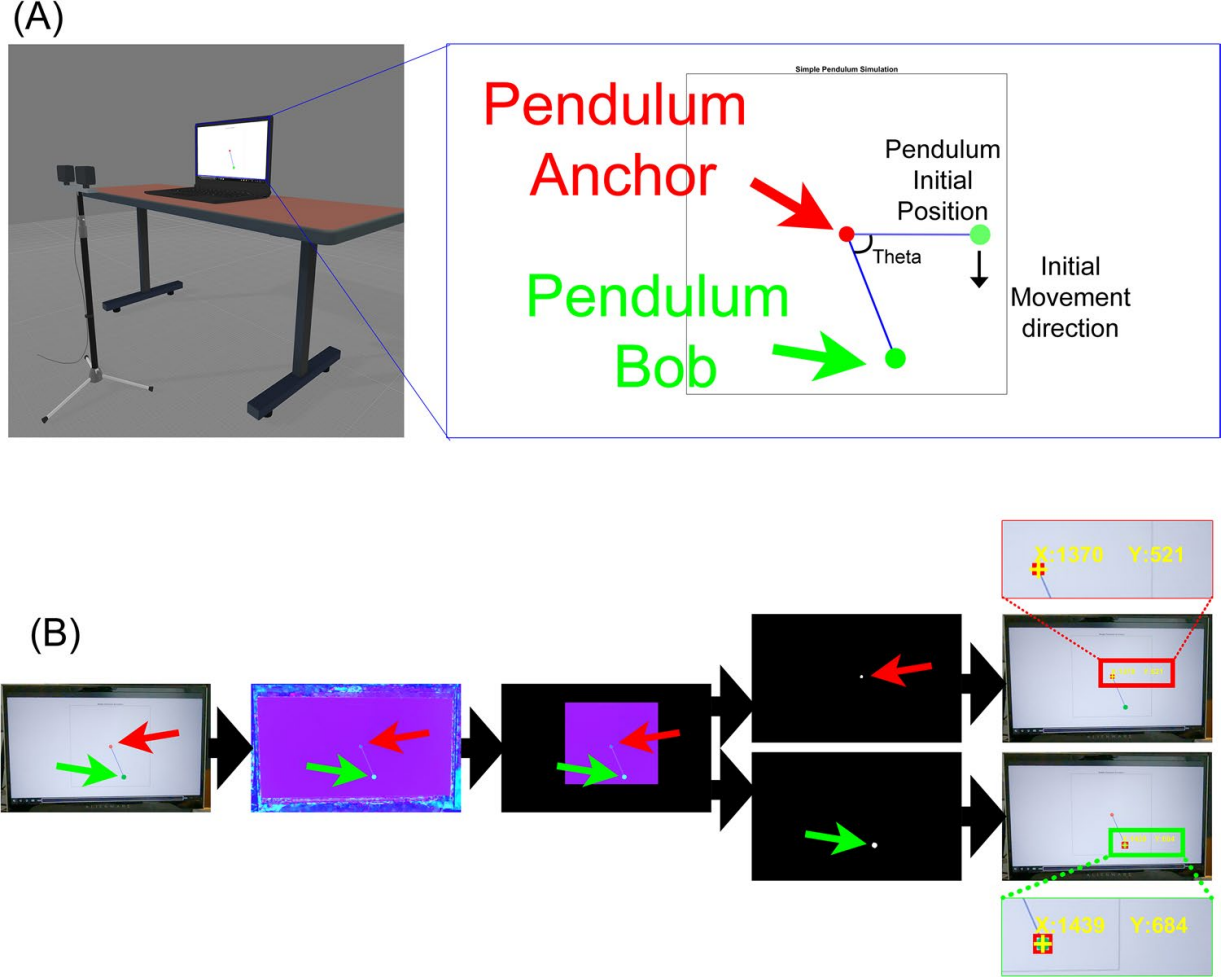
Experiment 2 setup and object detection process. (**A**) Experimental setup. The action cameras on the mount were placed in front of the laptop PC monitor. A simulated pendulum was played on a laptop 300 Hz fresh rate. The pendulum simulation includes the pendulum anchor (red circle), rod (blue line), and bob (green circle). The pendulum started at a pi/2 angle and played for 10 s. (**B**) Object detection process. Green and red circles were separately detected from the cropped HSV image.

A 10-s pendulum simulation video was created using a MATLAB custom script^26^. Detailed pendulum simulation settings are listed in Supplementary Table 3. In the pendulum simulation video, the pendulum bob was plotted as a green circle. The anchor of the rod (fixed point) was plotted as a red circle. The stereo camera system was placed at a 50 cm distance from a laptop PC monitor. Two action cameras were attached to a dual twin mount adapter with a 15 cm baseline distance. The video capture setup was the same as Experiment 1, but used two higher frame rates (60 and 120 fps) to examine the impact of the frame rate on the system's accuracy. To minimize the motion blur, the shutter speed was locked at 1/960th seconds (45°). The laptop PC monitor with a refresh rate of 300 Hz was used to present the simulated pendulum video (Fig. 3).

#### Stereo camera calibration

The same stereo camera calibration was performed, but the calibration images were captured around the laptop PC monitor.

#### Data acquisition

Video capture was automatically initiated using an audio file with a video capture initiation command, followed by a one-second whistling sound for video synchronization. Once the video capture was started, the 10-s pendulum simulation video was played. When the pendulum simulation video stops, the camera recording was manually stopped using a remote controller. This data acquisition was repeated with different frame rates.

#### Data analysis

The same calibration and video pre-processing procedures in Experiment 1 were used. The same object tracking procedure in Experiment 1 was also used. In this experiment, two objects were tracked: (1) the green-colored pendulum bob and (2) the red-colored anchor of the pendulum.

3-D positions of the pendulum bob and anchor were calculated using triangulation. Then the pendulum angle from the video data (theta_camera) was calculated using inverse kinematics from the 3-D positions of the pendulum bob and anchor. The theta_camera was compared to the ground truth angle data from the simulation (theta_simulation) and calculated the root mean square error (RMSE) of the theta_camera. Lower RMSE indicates more accurate pendulum angle estimation from the cameras. The pendulum simulation Cartesian coordinate positions in x (mediolateral) and y (superior-interior) directions from the camera were also compared to the ground truth pendulum Cartesian positions in x and y directions from the simulation. The pendulum rod length in the simulation data was scaled to that in the 3-D position data from the camera. We calculated the root mean square error (RMSE) for each direction.

The pendulum angles computed from two distinct video sets with various frame rates and the pendulum angle from the simulation data were compared using the one-way ANOVA test. Theta_camera data from videos at 60 fps were upsampled to 120 Hz to match the size of the data to theta_camera data from videos at 120 fps. The one-way ANOVA test was performed using the theta_camera data from videos at 120 fps (theta_camera_120), the upsapled theta_camera data from videos at 60 fps (theta_camera_60_resample), and the theta_simulation data at 120 fps. Significance level was set at 0.05. A custom MATLAB script was used for the statistical analysis. A multiple comparison test was also conducted to detect which of the theta data sets differed from the others.

### Experiment 3: Feasibility of arm and hand motor function test using the stereo camera system

We tested the feasibility of our stereo camera system to detect changes in fine hand motor skill after a practice in non-disabled young adults. Behavioral data from eleven non-disabled young adults in an ongoing clinical rehab pilot trial were used in this analysis. This research project was approved by SUNY Upstate Medical University Institutional Review Board (#1479630). We performed the research in accordance with relevant guidelines/regulations. We obtained the informed consents from all participants at the beginning of their participation. Individuals aged between 21 and 40 who had no neurological and musculoskeletal disorders were included. Individuals were excluded if they had any impairments that can affect their hand and arm motor function. Written informed consent was obtained from all participants. The demographics of participants are summarized in Supplementary Table 4. To summarize, all the individuals were right-handed. Eight (73%) of the participants had prior experience using chopsticks. All individuals reported poorer self-efficacy while using chopsticks to pick up a small object with their non-dominant hand (mean ± standard deviation [SD] Self efficacy = 29.18 ± 21.21 out of 100) than when using their dominant hand (mean ± SD Self efficacy = 69.36 ± 31.53 out of 100). We utilized a standard motor skill learning experimental design, consisting of baseline motor skill test (10 trials), motor skill practice (50 trials), immediate (10 min after practice) motor skill retention test (10 trials), and a 24-h delayed motor skill retention test (10 trials). To reduce the impact of past chopstick experience on motor skill acquisition, participants performed the motor task with their non-dominant hand. The participant's self-report of hand dominance was used to assess hand dominance. This experiment was conducted at the Motion Analysis Lab in the Human Performance Institute of the SUNY Upstate Medical University.

#### Experimental setup

**Figure 4:**
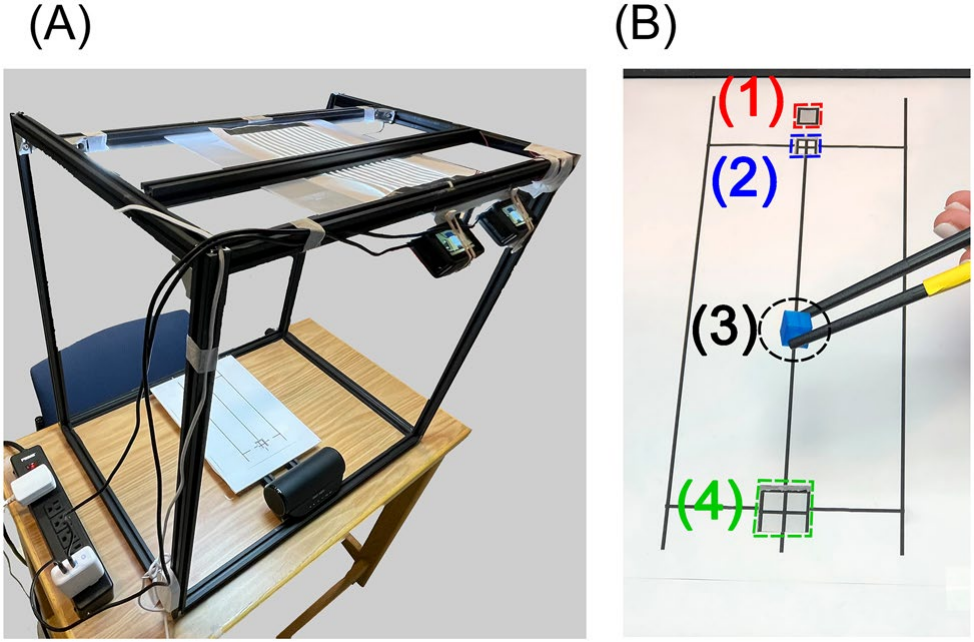
Experiment 3 setup. (**A**) Chopstick object pick-up test workstation. The workstation was constructed using aluminum extrusion profiles. On the back top side of the workstation, two cameras on a dual twin mount were installed. The cameras were positioned at a 30° angle from the table. The test template was positioned in the center of the table. The workstation's top was illuminated by an LED light. To provide auditory cues, a Bluetooth speaker was placed on the back side of the table. (**B**) The chopstick object pick-up test template. (1) Chopstick holding position. Participants positioned the tips of their chopsticks at this chopstick holding spot at the beginning of each trial. When a participant hears the whistle, they began moving the chopsticks to pick up the object. (2) Target object home location. The target plastic block is placed at this home position at the start of each trial. (3) Target object—blue plastic block. This blue-colored plastic block sized 1 cm on each edge. A 3-D printer was used to create this object. (4) Target location. The participant moves the plastic block from the home location to this location in 5 s for each trial. The target area measured 2 × 2 cm^2^. A trial was regarded successful if the participant correctly placed the plastic block in this area without dropping it within five seconds.

A workstation was built using aluminum profile extrusions. A template for the test was printed on letter-size paper and attached to a foam sheet. There was a groove on the target object's home position to hold the object. Also, there was a groove on the target location. The sheet was placed on the table in the center of the workstation. (Fig. 4) The stereo camera system was attached to the top rear part of the workstation. A White LED light was attached to the top center of the workstation (Fig. 4).

Two action cameras were attached to a dual twin mount adapter with a 15 cm baseline. The angle between the camera plane and the template sheet was set at 30°, and the template sheet was located about 0.4–0.6 m from the left camera. Details of the video capture setting are listed in Supplementary Table 2.

The same stereo camera calibration was performed, but the calibration images were captured around the template sheet.

#### Experimental procedure

Following the informed consent procedure, individuals were assessed to see if they were eligible for the experiment. After the screening and informed consent, the participants completed the baseline chopstick object pick-up test (COPT). The COPT was developed to evaluate chopstick operation skill. The participant sat on a chair and was positioned in front of the workstation. The participant held a pair of chopsticks with their non-dominant hand and placed the tips of chopsticks on a designated point on a template sheet. An audio file was played using a Bluetooth speaker to trigger the video capture and provide a participant cue. The audio file played the 'GoPro Start Recording' sound to initiate the video capture. Then the file played the 'Ready' sound with a 0.5-s whistle sound. When the participant heard the auditory cue (whistle sound), the participant picked up a plastic block (1 cm on edge) from a designated home location using a pair of chopsticks. The participant then moved the block to a target location 20 cm away and placed the block as near to and soon as possible in the middle of the target position. The foam sheet contains a rectangle groove at the object's home location to keep the block in place. The target spot has a rectangular groove (2 × 2 cm^2^). The trial is considered unsuccessful if the participant drops a block before it reaches the target area. It is also regarded a failed trial if the participant cannot transfer the object to the target area in five seconds. An investigator relocated the plastic block to its original location after each trial. There was a 10- to 20-s rest between each trial. The participant performed three familiarization trials followed by ten actual trials for the baseline test. If the participant had never used chopsticks previously, the investigator demonstrated and instructed how to hold the chopsticks. The video recording was ended automatically 5 s after the auditory cue using verbal command. Each trial footage was kept in its own video file.

Following the baseline COPT, participants conducted the chopstick object pick-up task practice. The practice consists of ten blocks, each with five trials (50 trials in total). After that, participants completed an immediate retention (IR) COPT 10–15 min later. Participants also completed a delayed retention (DR) COPT 24 h later. Ten trials of the chopstick object pick-up task were included in each retention test.

#### Data analysis

The same calibration and pre-processing procedures in Experiments 1 and 2 were used. In addition to the video data pre-processing, the timing of the auditory cue for movement initiation was identified using audio data in both videos. A custom MATLAB script was used to determine the timing of the whistle sound in the video data. The audio data processing included rectification, smoothing, filtering, and linear enveloping. The peak of the audio data, which shows the peak amplitude of the audio data for the whistle sound, was then detected. This auditory cue timing information was used to calculate temporal kinematic variables.

**Figure 5:**
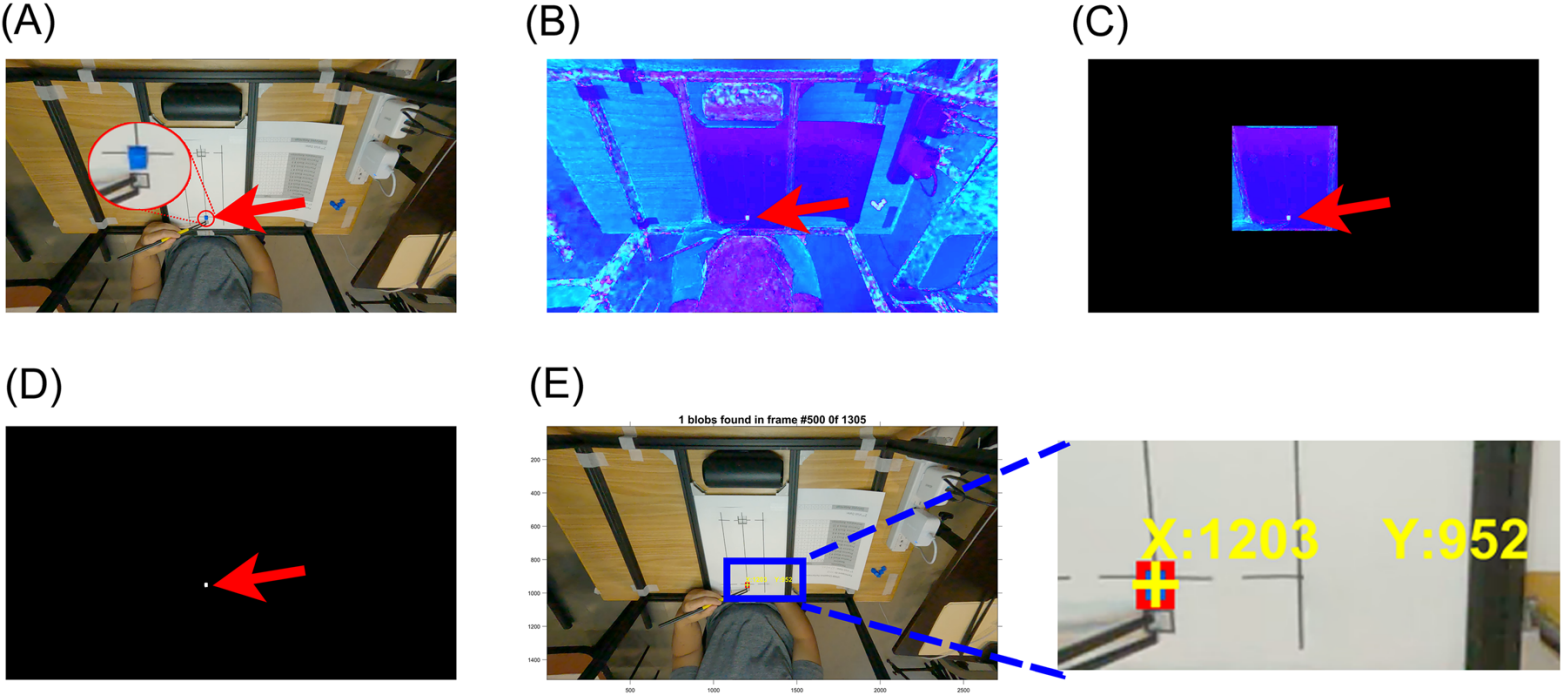
Experiment 3 object detection process. (**A**) Raw image in RGB color space. (**B**) Image in HSV color space. (**C**) Cropped HSV image. (**D**) Binary mask for the detected target object. (**E**) The final result of the object detection with bounding box (red square) and centroid (yellow cross). The Cartesian coordinate of the centroid is displayed in yellow text. The pixel was the unit of the centroid's Cartesian coordinate.

The same object tracking procedure in Experiments 1 and 2 was used, but different target HSV color ranges were used to detect the blue plastic block. (Fig. 5).

For the kinematic analysis, we utilized a custom MATLAB script to analyze the kinematic data and calculate kinematic metrics. 3-D positions of the block were calculated using the 'triangulate' function with the centroid positions of the object from two video data. The 3-D position data were rotated on mediolateral axis 30° clockwise and reoriented the object's home location as a new origin. This process was because the origin of the 3-D position data from the triangulation is the center of the left camera plane. Then we calculated the resultant position in x (mediolateral), y (superior-inferior), and z (anterior–posterior) axes. The resultant data were filtered using a 3rd order Butterworth filter with a cutoff frequency of 3 Hz. Tangential velocity was calculated as the first derivative of the filtered resultant. Movement onset, peak velocity, and movement offset of the object were automatically determined using the velocity profile. Movement onset was defined as the first frame of the block movement, where the tangential velocity is above 10% of the peak velocity^2^. Movement offset was defined as the last frame of the block movement, where the tangential velocity is above 10% of the peak velocity^2^ (see Supplementary Fig. 6). An investigator (BK) visually inspected the individual trial movement onset and offset to determine these were correctly labelled. The peak velocity was defined as the maximum tangential velocity amplitude of the trial that exceeds the amplitude of 0.3 m/s. We calculated the following temporal and spatial kinematic variables using the movement onset, offset, and peak velocity: Object Grasping Duration (GD)—Time difference between auditory cue and block movement onset; Object Moving Duration (MD)—Time difference between block movement onset and offset^1,2,27^; Total Performance Duration (PD)—Sum of GD and MD; Peak Velocity Amplitude (PV)—Highest velocity during each trial^1,2,27^; Log Dimensionless Jerk (LDJ)—a measure of the movement smoothness calculated from the third derivative from the resultant of the position^28^.

To compare kinematic variables across different time points (baseline, practice, immediate retention, and delayed retention), a linear mixed-effects regression analysis on each kinematic variable was performed. We adopted linear mixed effects regression analysis over traditional repeated measures ANOVA for two reasons: (1) to improve statistical power; and (2) to prevent data imputation owing to missing data or an unequal number of trials between time points^29^. The linear mixed effects regression can be used to reduce false-positive correlations caused by sample structure and to increase statistical power^30^. Because the data analysis only included successful trials, each participant had a different number of successful trials for each time point. The variability in the number of successful trials between participants and within participants may have an impact on the statistical results. The linear mixed effects regression analysis using the participant's random effects term can reduce the impact of an uneven number of successful trials within and between individuals. In the linear mixed effects regression analysis, the timepoint variable was an independent categorical variable, and each kinematic variable was a continuous dependent variable. The timepoint variable was the fixed-effects predictor variable, and participant ID number and group allocation were random-effect terms in the model. The ID random-effects term was only for the intercept, and the group random-effects term was for both the intercept and the timepoint variable.

## Results

### Experiment 1

**Figure 6:**
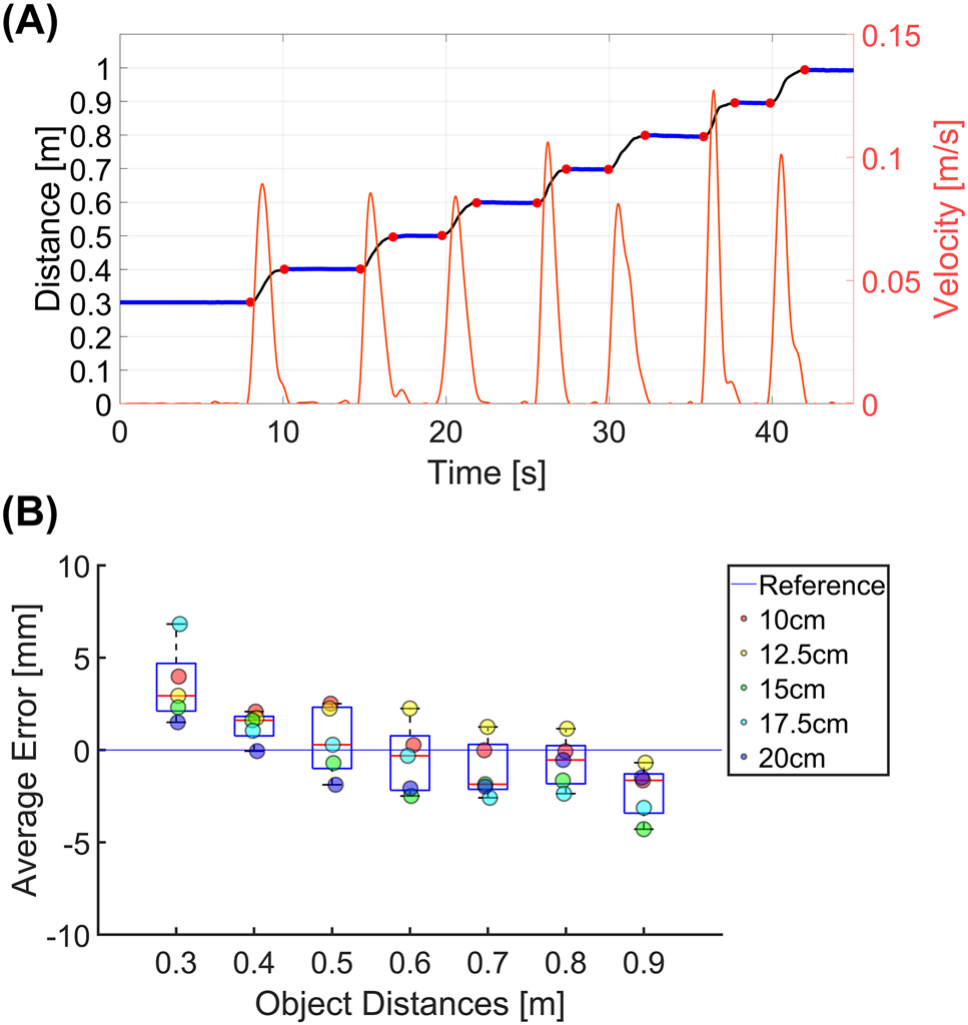
Experiment 1 results. (**A**) An example result from a 15 cm baseline setting. On the left y-axis, the distance between the left camera and the object is shown. On the right y-axis, the tangential velocity profile is shown. Blue lines indicate when the green ball was not moving. Black lines indicate when the green was moving. Red dots indicate the movement onset and offset of the green ball based on the velocity profile. (**B**) Average distance estimation error at different distances and baselines. Boxplot indicates the distance estimation error distribution at each object's distance from the camera. The Scatter plot indicates the distance estimation error at different baseline settings.

The 3-D position the green ball object was successfully estimated from the stereo camera video data. The green ball movement was segmented as the moving and the stationary portions using the tangential velocity of the object movement. (Fig. 6A).

The absolute distance estimation errors across different baseline distances were less than 5 mm. There was no statistically significant difference in the distance estimation error among different baseline distances (χ^2^(4) = 9.35, p = 0.053). There was a significant difference in distance estimation error among different object distances (χ^2^(6) = 18.46, p = 0.005). The absolute estimation error was minimal when the distance between the object and the camera was between 0.4 and 0.7 m. (Fig. 6B).

### Experiment 2

**Figure 7:**
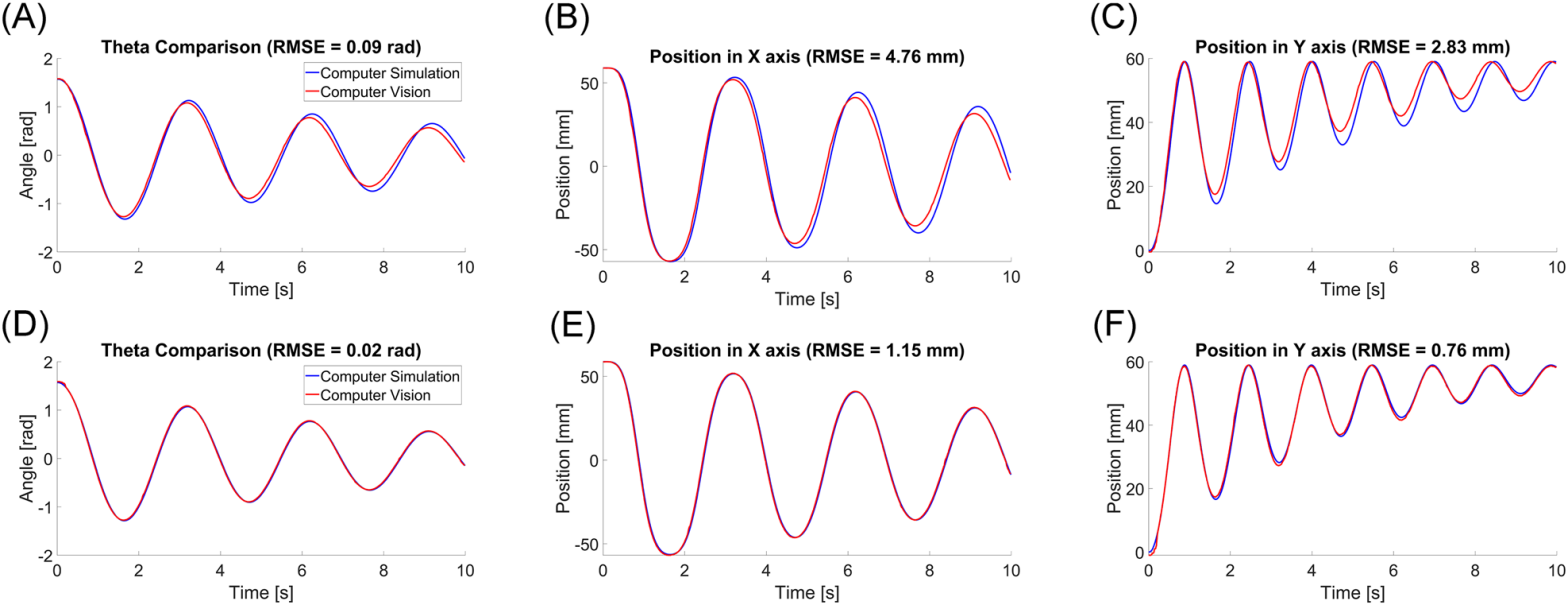
Experiment 2 results. (**A**–**C**) Data from 60 fps of frame rate. (**D**–**F**) Data from 120 fps of frame rate. (**A**,**D**) Pendulum angle comparison. (**B**,**E**) Position in x (mediolateral) direction comparison. (**C**,**F**) Position in y (superior-inferior) direction comparison.

The pendulum angle was successfully estimated from the stereo video data. The root mean square error (RMSE) of the estimated theta was 0.09 and 0.02 rad at 60 fps and 120 fps, respectively. (Fig. 7A,D) With a frame rate of 120 fps, the RMSE of the estimated position of the pendulum at the x- and y-axis were 1.15 and 0.76 mm, respectively. With a frame rate of 60 fps, the RMSE of the estimated position of the pendulum at the x- and y-axis were 4.76 and 2.83 mm, respectively. (Fig. 7) The one-way ANOVA was conducted to compare the effects of video frame rates on pendulum angles (theta) estimation. There was a statistically significant effects of the video frame rates on estimation of the pendulum angles (F(2, 3602) = 8.79, p < 0.001). The theta estimated from videos at 60 fps was statistically significantly different from the theta estimated from videos at 120 fps and the theta calculated from the simulation data (p < 0.001). There was no statistically significant difference in theta between the video data at 120 fps and the simulation data (p = 0.978).

### Experiment 3

**Figure 8:**
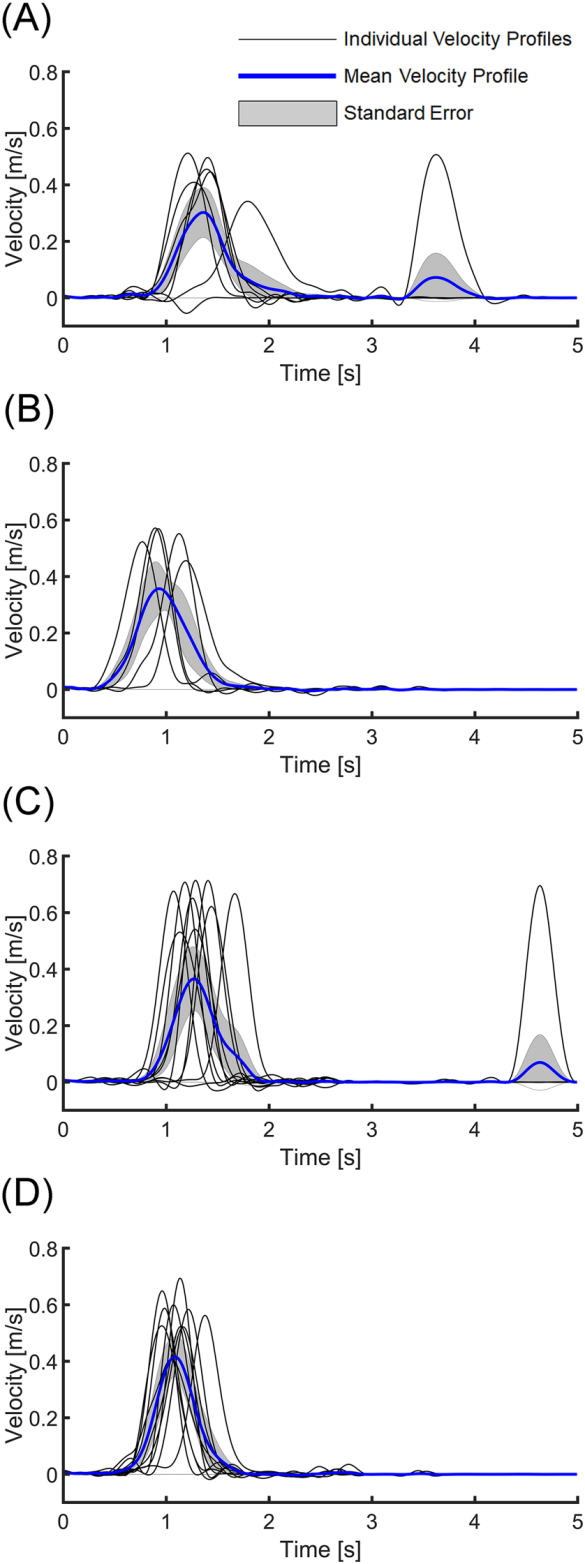
Exemplar tangential velocity profiles in different time points from a participant. (**A**) Tangential velocity profiles of the object movements at baseline test (7 successful trials out of 10 baseline trials are presented). Each thin black line indicates a velocity profile of a trial. A thick blue line indicates the mean velocity profile of the timepoint. Gray shade indicates the standard error. (**B**) Tangential velocity profiles of the object movements at the fifth block of practice (5 trials). (**C**) Tangential velocity profiles of the object movements at immediate retention test (10 successful trials out of 10 baseline trials are presented). (**D**) Tangential velocity profiles of the object movements at delayed retention test (9 successful trials out of 10 baseline trials are presented.). Refer to Supplementary Fig. 7 for further information about the success rate of the chopstick motor skill trials.

The object's 3-D positions were successfully estimated from the stereo video data. Kinematic metrics of the chopstick object pick-up performance were calculated from the 3-D positions of the object (Fig. 8). At a group level, object movement duration (MD) was statistically significantly shorter for delayed retention timepoints than the baseline (t = − 2.44, p = 0.02). The total performance duration (TPD) was also significantly shorter for delayed retention than the baseline (t = − 2.38, p = 0.02). Peak velocity amplitude (PV) was also statistically significantly greater for practice (t = 2.82, p = 0.005) and delayed retention (t = 4.02, p < 0.001) timepoints than the baseline. The object grasping duration (GD, F(3, 784) = 2.04, p = 0.11), log dimensionless jerk (LDJ, F(3, 784) = 0.94, p = 0.42), and object trajectory length (F(3, 784) = 1.39, p = 0.25) did not show statistically significant differences among different time points. (Supplementary Fig. 5).

## Discussion

We developed a computer vision-based motion capture system using action cameras that can be used to assess fine hand motor skills with object manipulation. Several different experiments demonstrated the system's accuracy and feasibility in detecting and tracking an object manipulated by a hand, reconstructing 3-D positions of the object, and estimating kinematic metrics of the object's movement to represent fine hand motor skills.

Our experiments demonstrated that the object can be accurately detected and tracked based on its color using two high-resolution and high-frame rate action cameras. From our experiments, we have learned that several factors should be considered to accurately track the moving object in the video using computer vision technology. First of all, the light source should provide bright light to the workstation. In a low light condition, the object color is not differentiated from the background, leading to object detection failure. One solution for the low light condition would be to increase the camera's ISO max setting^31^. Although a higher ISO setting allows the recording of the object in a low light environment, this setting will also increase the sensitivity of the camera sensors, which means more noise in the video data^31^. Thus, it will lead to a less accurate object tracking.

Frame rate is another vital factor in improving the accuracy of the system. The frame rate should be high enough for faster movements, such as ball throwing in sports^31^. Specifically, most daily activities related to goal-directed arm reaching would require a high frame rate to estimate the movement kinematics accurately. As we demonstrated in Experiment 2, the RMSE of the pendulum angle estimation was greater for 60 fps than 120 fps. This result indicates that a higher frame rate is required to reduce the estimation error in faster movement. However, from a practical standpoint, higher frame rates would be computationally expensive. A 120-fps video would have twice as many frames for any given video capture period as a 60-fps video. In terms of the computational efficiency of the data analysis, a low frame rate would be more feasible. However, if the target movement is fast, such as sprint, baseball pitching, etc., the low frame rate would increase the estimation error of the kinematic measures.

Shutter speed is also critical to reducing motion blur. We used an automatic shutter speed setting during our preliminary data collection for Experiment 3. The automatic shutter speed setting will select the slow shutter speed to maximize the light reaching the sensor^31^. Thus, the automatic shutter speed setting will increase motion blur. In our preliminary experiment, the slow shutter speed generated motion blur even during the chopstick object pick-up task, which does not require fast arm reaching. Several computational methods reduce motion blur or track an object even with motion blur, but it would require additional step in the object detection and tracking, which would increase the data analysis time. We minimized the motion blur by using a faster shutter speed (i.e., low lock shutter angle). One caution would be that the faster shutter speed means less light entering the camera sensors. Therefore, there is an advantage to having a well-lit capture volume when using a fast shutter speed.

Our experiments used 120 fps of frame rate and 1/960 s shutter speed. Based on a recent review article that detailed the camera setup for motion analysis in sports science^31^, these video recording settings are sufficient to record kinematics of the object manipulated by a hand. Pueo recommended high-speed camera settings based on the velocities of sports projectiles^31^. In the recommendation, basketball had the slowest velocity of the projectiles among selected sports in the review. The author recommended at least 48 fps and 1/250 s shutter speed to record the basketball projectiles. Given that object movement during the chopstick object pick-up test is much slower than the basketball projectiles, our camera settings were sufficient to record the object movement without motion blur and perform frame-by-frame analysis. Pueo also provided equations to calculate the minimum frame rate and shutter speed. For the chopstick object pick-up test, the maximum peak velocity of the object movement was about 0.8 m/s, and the maximum distance of the object movement between consecutive frames would be 2 cm. Based on Pueo's equation, the minimum frame rate to capture the object movement would be 40 fps (object velocity/maximum distance of the object between two frames). Our preliminary data showed that motion blur is one of the most critical barriers to color-based object detection. Thus, shutter speed is critical to eliminate the motion blur. The maximum motion blur would be 1 mm, which is 1% of the target object size, to eliminate motion blur in the chopstick object pick-up test. The minimum shutter speed for the chopstick object pick-up test would be 1/160 s (object velocity/maximum motion blur) when we allow the 1 mm of maximum motion blur.

Based on these calculations, a lower frame rate and slower shutter speed can be used to record the object movements during the chopstick task, which would be more practical in terms of computational cost for data analysis.

The angle between the camera plane and the target object plane is also essential to improve the object tracking accuracy. An experiment was performed to determine the angle between the camera plane and the object plane (see Supplementary document). The angle between the camera plane and the object plane should be less than 75°. If the angle is more than 75°, it increases the estimation error of the object's position (Details on the additional experiment can be found in the Supplementary document). An appropriate angle between the camera plane and the target movement plane reduces distortion of the image. Specifically, distortion of the image would be the least at the center of the camera view and highest at the edges. The greater angle between the camera plane and the object plane would locate the target object closer to the edge of the camera frame and thus have more distortion. For our experiment, the angles between the camera plane and the target movement plane were set at 0 (Experiments 1 and 3) or 30 degrees (Experiment 3) to minimize the image distortion. It would be ideal for maintaining a 0° angle between the camera and the target object's movement plane. However, it may not be practical in all situations, such as when a target object may be occluded by the participant's hand. This is the case during our object pick-up test using chopsticks, and thus to avoid object occlusion, a 30° angle was ideal.

Another vital camera setting for the system's accuracy would be the video resolution^17^. Zago et al.^17^ reported that the increased video resolution improves the accuracy of the human pose estimation using two full-HD webcams. They stated that higher video resolution can decrease the uncertainty in the body landmark identification on the image frames, which leads to more accurate 3D reconstruction results^17^. In our experiment, we utilized a high-resolution video capture setting (2704 × 1520 pixels) in this study, which is two times as many pixels as the high definition (HD) resolution (1920 × 1080 pixels). Higher video resolution improves landmark identification but requires higher computational power to process the high resolution videos, specifically high-end graphic card. Previous computer vision-based markerless motion capture studies have utilized HD or standard definition (SD) resolution (720 × 480 pixels). SD resolution would be sufficient for object tracking in our experiments. However, it is possible that tracking a small object, like the blue block (1 cm on edge) in Experiment 3, in SD resolution videos would be less accurate, leading to false 3D reconstruction results. Since processing higher resolution video is more computationally expensive, the video resolution should be determined based on the available resources (high functioning computer) and the target object size.

Previous studies using a stereo camera-based markerless motion capture system have suggested that baseline distance (distance between two cameras) is another crucial factor for the depth estimation accuracy at different distances. For example, Zago et al.^17^ showed that increasing the baseline distance improved the 3D reconstruction accuracy of the whole-body joint positions during gait. They suggested a 1.8 m baseline for assessing gait. In our experiments, however, the workspace for the fine hand motor skill was much smaller than the workspace for the gait analysis experiment. In our experiments, the amplitude movement of the target object was also not as big as gait or other lower extremity activities. Thus, we used a short baseline distance—0.15 m compared to other settings for lower extremity or whole-body motion capture for gait analysis. Our Experiment 1 demonstrated that the baseline distance is not a factor in the depth estimation accuracy when the object is located between 0.3 and 1-m distance from the camera. We used a 0.15-m baseline for a practical reason. If the baseline is too short, the disparity between the two cameras would be less, leading to a greater 3-D reconstruction error. If the baseline is too long, the cameras cannot capture the whole calibration images within a 0.5 m distance.

In Experiment 3, we tested the feasibility of our system to assess fine hand motor skills during an object manipulation using a pair of chopsticks. We compared motor performance at different time points using a standard motor skill learning experimental design. We demonstrated that participants showed statistically significant improvements in motor performance between the baseline and immediate retention tests at the group level. Further, they also showed motor skill learning between the baseline and the delayed retention tests. We utilized kinematic measures of the object movements instead of the upper extremity joint kinematics. In this object pick-up task using chopsticks, the object kinematics represent the endpoint kinematics of the motor performance. These results indicate that the proposed markerless motion capture system can assess fine hand motor performance by tracking the object manipulated by a hand. We were able to demonstrate that our system can assess the improvement in performance time of a fine hand motor skill. This approach would be more feasible for clinical and research purposes, as this approach is less computationally expensive than tracking human pose estimation and joint tracking. Instead of tracking the body segments, we tracked an object manipulated by a hand using chopsticks.

We utilized the object pick-up task using a pair of chopsticks with a non-dominant hand. This task requires a high level of hand dexterity^32–34^. Previous studies have used similar object pick-up tasks using chopsticks to assess hand dexterity^35^ or as an intervention to improve hand function for people with neurologic disorders^33,34,36^. Several previous studies have utilized the chopstick skill tasks for motor skill acquisition. Bosch and colleagues employed a chopstick skill task to determine how individuals learn a novel and challenging tool use^37^. Their chopstick skill task was to pick up a marble and lift it to the top of a cylinder repeatedly as many as possible for a minute. Participants performed eight training sessions over 4 weeks. They found that participants improved their performance after training sessions. The performance improvement included a shorter time to grasp a marble and lift and drop the marble. These temporal variables are similar to our study. Our experiment calculated the time to grasp the block (GD) and the time to move the block to the target area (MD). Our participants did not show statistically significant improvements in GD, but did show improvements in MD. Given that participants only had one session of chopstick skill practice with 50 trials, the 50-trial practice may be insufficient to improve the GD. This result indicates that participants improved in controlling chopsticks while transporting the object. This result is consistent with Bosch and colleagues' findings that their participants improved the time to lift and drop marble (moving it from the bottom to the top).

Our kinematic data from Experiment 3 also showed improvement in object movement peak velocity after the practice. These results are partially consistent with previous findings from Sawamura et al.^34^. They found people showed improvement in speed and smoothness of the chopstick skills with their non-dominant hand after a 6-week training in chopstick operation with the non-dominant hand. In their experiments, the task was to move various objects, such as beans, marbles, and sponges, from a plate to another plate using a pair of chopsticks with their non-dominant hand. We did not observe increase in movement smoothness after the practice. This may be related to the fact that we only had one practice session, whereas Sawamura and colleagues investigated the effects of long-term chopstick training (i.e., 6 weeks) on chopstick movement smoothness.

In Experiment 3, participants also retained their improvements after 24 h, indicating motor skill learning as we expected. A novel motor skill acquisition requires motor memory consolidation, a process that occurs immediately after practice and during sleep^38^. Therefore, retention of the improvements in kinematic variables can represent the chopstick motor skill learning. These kinematic results from Experiment 3 indicate that this computer vision-based motion capture system can be employed to assess fine-hand motor skills improvement. Using the motor skill learning study design, we confirmed the feasibility of this system to assess improvement in fine hand motor skills by tracking the manipulated object and calculating the kinematic metrics of the object movements.

There are a few limitations of this study. First, tracking an object instead of upper extremity joints does not provide *how* the participant performs the task^39^. For instance, chronic stroke survivors would employ compensatory movement strategies, such as excessive trunk forward displacement or shoulder abduction, to substitute the lack of elbow extension and shoulder flexion during arm reaching to transport the object to the target location^40,41^. It would be difficult to determine the motor control strategies to complete the task without tracking the upper extremity joints. Our markerless motion capture system, however, has the capability of recording upper extremity joint kinematics. We can record the upper extremity joint and object motions by changing the camera angle and lens settings. This combined approach will allow us to quantify the motor performance and motor control strategies together. One caution would be that performing the markerless motion tracking of upper extremity joints is computationally expensive, which means that the human pose estimation and tracking in 3D requires an expensive high-performance computer.

Another limitation of this study is that the video data collection and analysis cannot be performed in real-time. The cameras utilized in this study were off-the-shelf products available online. These are affordable cameras capturing high-resolution videos (up to 5 K resolution) with high frame rates (up to 240 fps). Computer vision cameras with real-time data collection and analysis are costly and have limited resolutions and frame rates. The limited resolution and frame rate for real-time data collection and analysis would be due to the high video file size. In Experiment 3, the video data were acquired at 2.7 K resolution and at 120 fps of frame rate. A 5-s video for one trial of the task from one camera was about 130 megabytes (MB) in size. Real-time video data collection and analysis for high resolution and frame rate video data would be more computationally expensive and complex to achieve with currently available video cameras.

Video data analysis for Experiment 3 took about 2.5 min on average with a high-end laptop PC (Intel^®^ Core™ i9-10980HK CPU, 32 GB RAM, NVIDIA GeForce RTX 3080 with 16GM GDDR6) and 3.7 min on average with a regular laptop PC (Intel^®^ Core™ i7-1065G7 CPU with integrated Intel^®^ Iris Plus Graphics, 8 GB RAM) for each pair of the video data for each trial. Given that there was a total of 80 trials, the video data processing time was inefficient (video data analysis took about 200 min per participant using a high-end laptop PC). Therefore, data analysis requires a high computational cost, even using this simple method to detect and track an object. This approach was computationally expensive because we calculated the 3-D positions of the object movements. Some clinical applications may not require 3-D kinematics. In that case, we may use just a single camera to calculate some kinematic variables in 2-D. The 2-D kinematic analysis will require less computational cost and be more feasible for clinical usage. Further, reducing the resolution and the frame rate to cut down the computational cost for data analysis is possible^31^. Lastly, if the task involves manipulating a larger object, reducing the video resolution is another solution to reduce the computational cost of data analysis^17^.

In addition, we did not use the passive marker-based optical motion capture system as the gold standard for kinematics measurement. We did not employ the conventional marker-based motion capture approach primarily due to technical limitations of the marker-based system. We tracked the movement of a small item in our experiments. The marker-based system we have in our lab employs marker triads, which are too large to attach to the target object. For example, in Experiment 3, we utilized a plastic block that measured 1 cm on the edge. If we attach the passive marker triad to this object, it will be invisible to the camera, preventing the stereo camera system from recording the whole view of the object. Furthermore, the marker triad will make it more difficult to pick up the plastic block with a pair of chopsticks. We used more precise ground truth measurements instead of the marker-based motion capture approach. We used the tape measure to estimate the distance between the camera and the object in Experiment 1. We employed a computer simulation of a pendulum in Experiment 2, and the kinematic data calculated from the computer simulation data served as a ground truth measure. As a result, we consider that utilizing ground truth measures rather than the marker-based motion capture system is a better way to assess the accuracy of the proposed motion capture system.

Lastly, we only had eleven non-disabled young adults in Experiment 3 to test the feasibility of the system to assess fine hand motor skills. To increase statistical power, we used linear mixed effects regression analysis. Instead of utilizing a typical repeated measures ANOVA using the average data of each time point for each individual, we performed the linear mixed effects regression using individual trial data for each participant. This statistical approach would compensate for Experiment 3's small sample size, and also reduce the effects of individual variability in their baseline chopstick skill performance.

## Conclusion

Markerless object tracking using two action cameras is an accurate and feasible approach to assess fine hand motor skills by recording the kinematics of the manipulated object. This approach will allow researchers and clinicians to assess fine hand motor skills more objectively and accurately using affordable and commercially available cameras. We need further studies to test this system to assess upper extremity motor performance and motor control strategies using this system in patient populations.

## Supplementary Information

Supplementary Information.

## Data Availability

Deidentified data analyzed in this study will be available to share upon request to the corresponding author.
