# Supplementary material for: Accuracy and feasibility of a novel fine hand motor skill assessment using computer vision object tracking
Source: Sci Rep. 2023 Feb 1;13:1813. doi: 10.1038/s41598-023-29091-0 (PMC9892571; doi:10.1038/s41598-023-29091-0)
Supplement: Supplementary file 1 — Supplementary Information. [file 41598_2023_29091_MOESM1_ESM.pdf]

# Accuracy and feasibility of a novel fine hand motor skill assessment using computer vision object tracking

# Supplementary Materials

## Additional Experiment: Validation of the stereo camera system accuracy for estimation of the distance between objects on a transverse plane in still images

### *Setup*

Black squares (2 X 2 $cm^2$) with a white cross in the center were printed on paper. (Supplementary Figure 1) A total of 20 squares (4 in each row X 5 rows) were printed with 5 cm center-to-center distance between squares in the anterior-posterior and medio-lateral directions. Two GoPro Hero 9 cameras were attached to a dual twin mount adapter with a baseline of 15 centimeters between two cameras to create a stereo camera system. (Supplementary Figure 1-A) The dual twin mount adapter was designed with TinkerCad software and 3D printed using a FlashForge 3D Printer. The mount 3D design is available online. Cameras were set to record videos at 2.7K resolution (2,704 X 1,520 pixels) and 120 frames per second (fps). Other details of the camera settings are listed in Supplementary Table 1.

### *Stereo Camera Calibration*

The stereo camera system calibration was performed using a checkerboard pattern. The checkerboard pattern was printed and placed on a hardboard. The black and white checkerboard pattern was moved within the camera angles at a distance between 0.3 and 0.7 meters, and the stereo camera system captured images at 120 fps. For best calibration

accuracy, pattern images were captured at a distance similar to the distance from the camera to the printed squares. Further, the angle between the checkerboard and the camera plane remained less than 45 degrees.

Calibration videos were imported to Matlab, and videos were synchronized using audio data. Then every 50 frames were exported to a JPEG image file from each video data. This procedure generates approximately 100 calibration images for each camera. The calibration was performed using the Matlab Stereo Camera Calibration Toolbox. We performed recalibration without image pairs having reprojection errors greater than 0.6 pixels. (Supplementary Figure 2.)

**Supplementary Table 1. Camera Angle Validation Experiment Camera parameters**

| Parameters | Camera Angle Validation |
|---|---|
| Resolution | 2.7K |
| Frame Rate (fps) | 120 |
| Lens | Linear |
| Protune color | Flat |
| Bit rate | High |
| White balance | 5,000K |
| ISO minimum | 100 |
| ISO maximum | 1,600 |
| EV compensation | Not set |
| Sharpness | Medium |
| Lock Shutter (Shutter Speed) | 45° (1/960 s) |
| The angle between the camera sensor plane and target plane | 30°, 45°, 60°, 75° |
| Stereo Camera Baseline Distance | 15 cm |
| Distance between the camera 1 and the target object | ~ 60 cm |

## *Data Acquisition*

The paper with planar rectangle images was placed on a table. We mounted the stereo camera system on a tripod and placed about 0.6 meters from the paper. To examine the effects of the angle between the camera and the planar image on the distance estimation error, we captured images at four different angles: 30, 45, 60, and 75 degrees. (Supplementary Figure 1-

C) We used an audio file to trigger the video recording of two cameras. The video was captured for 10 seconds – a total of 1200 frames from each camera were acquired.

## *Data Analysis*

Video data from two cameras were imported to a laptop PC and processed using a custom Matlab script. From each video data, 15 frames were randomly selected. Each image was undistorted using the stereo parameters from the camera calibration. Then, we transformed the undistorted image from RGB to black and white binary images, and a bounding box was created for each square. Then the centroid of the bounding box was calculated as an image point. (Supplementary Figure 3)

A 3-dimensional location of centroid image point pairs from two cameras returned as a 15-by-3 matrix. The matrix contains 15 locations of matching pairs of centroid image points from two stereo images. The three-dimensional (3-D) positions of squares were estimated using the 'triangulate' function. (Supplementary Figure 4) Distances between adjacent squares of each frame were calculated along with anterior-posterior and medio-lateral directions. Then the distance from the camera was compared to the ground truth distance. Distance error was calculated in millimeters.

A one-way ANOVA was performed to compare the effect of four different camera angles on distance estimation errors.

## *Results*

Squares were successfully detected from the stereo camera video data. The mean absolute estimation error of distance between rectangles in mediolateral (M-L) direction was 0.20, 0.18, 0.17, 2.77 millimeters for 30, 45, 60, and 75 degrees of camera angles, respectively. The mean absolute estimation error of distance between rectangles in anterior-posterior (A-P)

direct was 0.59, 0.62, 0.88, and 4.79 millimeters for 30, 45, 60, and 75 degrees of camera angles, respectively.

A one-way ANOVA revealed that there was a statistically significant difference in mean estimation error in the A-P direction between at least two angles ($F(3, 956) = 3.26$, $p = .02$). Tukey's HSD Test for multiple comparisons found that the mean value of A-P direction error was significantly different between 45 degrees and 75 degrees of camera angle settings ($p = .044$, 95% CI = [-6.96 -0.06]). There was no statistically difference in A-P direction estimation error between 30 and 45 degrees ($p = .99$), between 30 and 60 degrees ($p = .99$), between 30 and 75 degrees ($p = .07$), between 45 and 60 degrees ($p = .99$), or between 60 and 75 degrees ($p = .05$). There was no statistically significant difference in mean estimation error in the M-L direction ($F(3, 896) = 2.6$, $p = .05$). (Supplementary Fig 5)

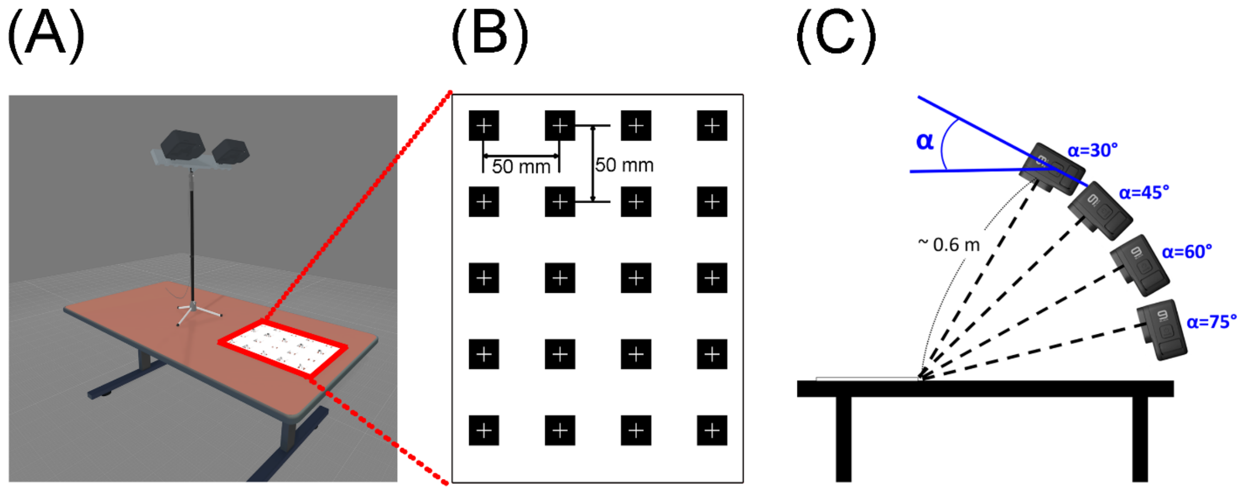


**Supplementary Figure 1. Experiment Setup for Angle Validation Experiment.** (A) Two action cameras were attached to a dual twin mount on a tripod. The camera pair was placed about 0.6 m from the target paper with squares. (B) Target paper with squares. Distance between adjacent squares is 50 millimeters in x (mediolateral) and z (anterior-medial) directions. (C) Four different camera angle settings. The angle (α) is between the camera plane and the table.

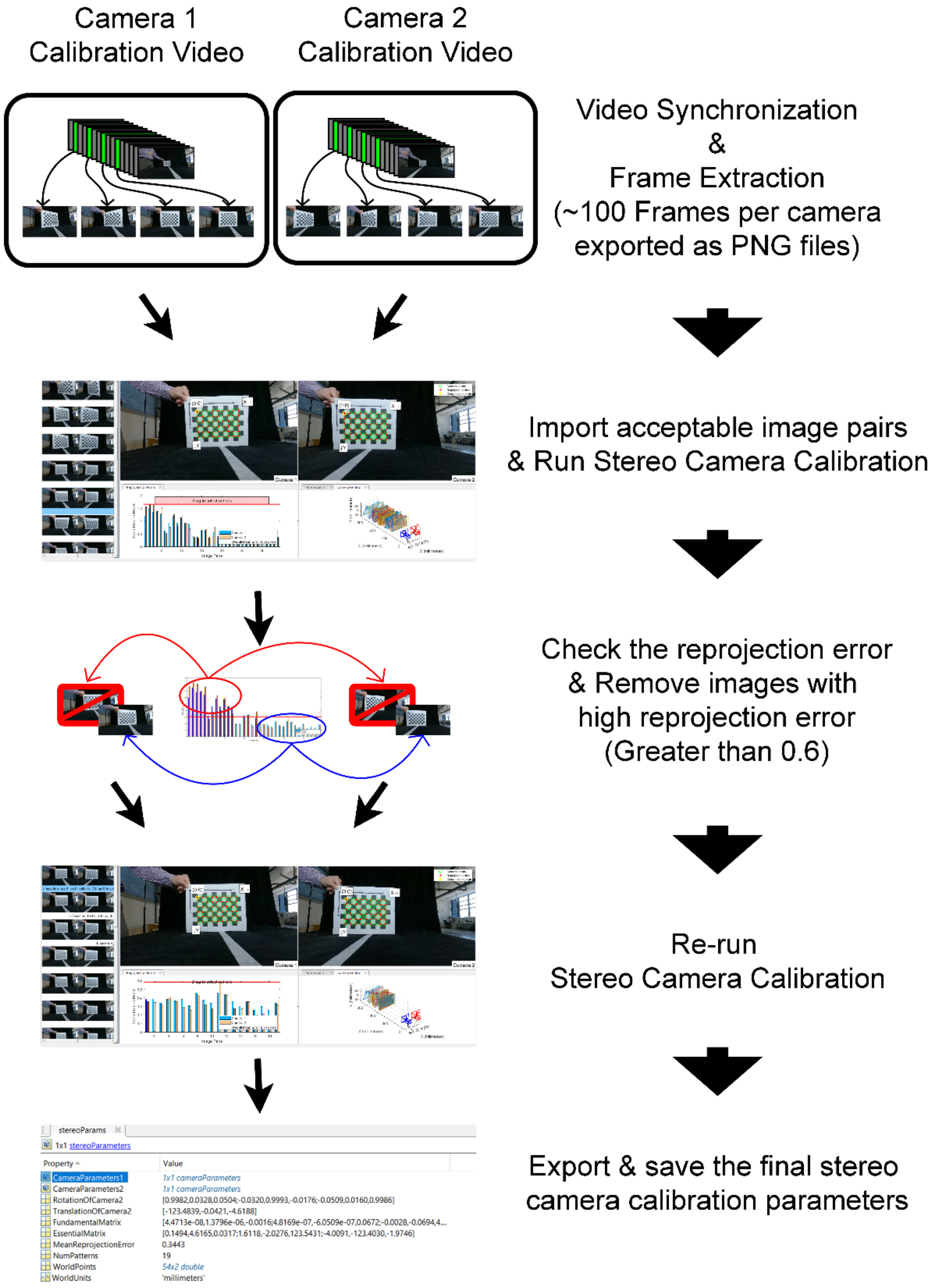


**Supplementary Figure 2. Stereo Camera Calibration Procedure.** Calibration images are from Experiment 1.

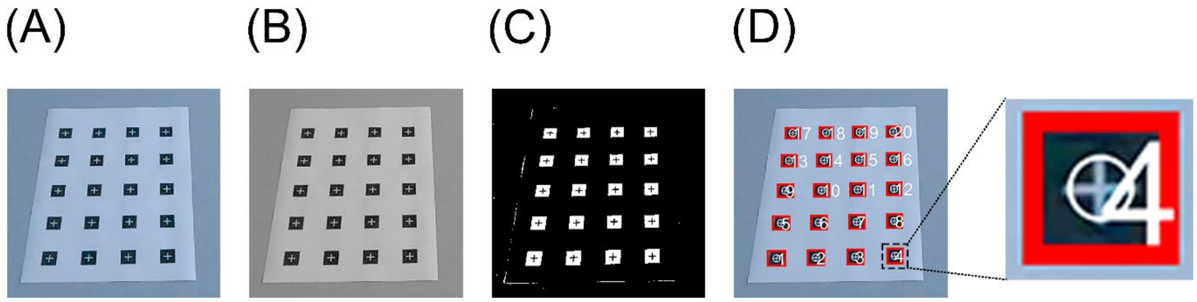


**Supplementary Figure 3. Experiment 1 square detection process.** (A) Raw image in RGB color space. (B) Image in grayscale. (C) Image in black and white. (D) Squares are detected with the bounding boxes (red boxes) and centroids (white circles).

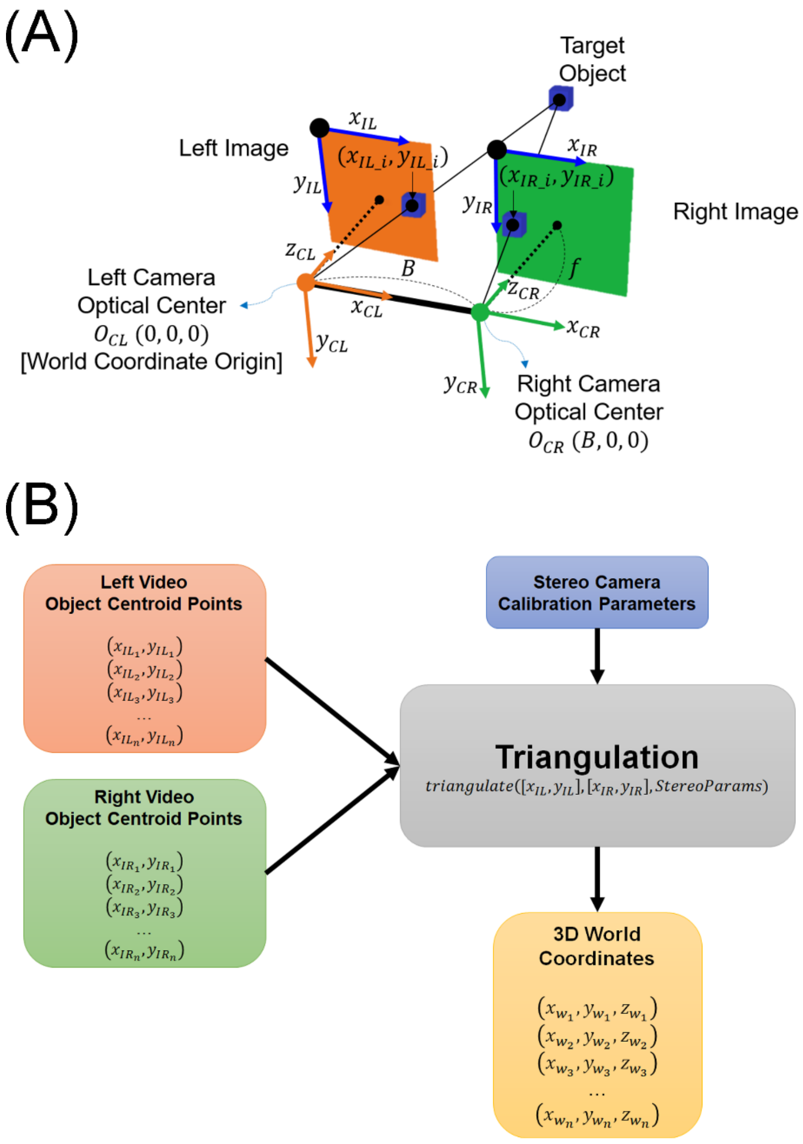


**Supplementary Figure 4. Estimation of 3D world coordinates of the object using triangulation.** (A) A geometrical model for the stereo camera system. $x_{CL}$, $y_{CL}$, $z_{CL}$: Left camera coordinate system; $O_{CL}$: Optical center of the Left camera. The $O_{CL}$ was an origin of the output 3D world coordinates of the object; $O_{CL}$: Optical center of the Right camera; $x_{CR}$, $y_{CR}$, $z_{CR}$: Right camera coordinate system; $x_{IL}$, $y_{IL}$: Left image coordinate system in the pixel; $x_{IR}$, $y_{IR}$: Right image coordinate system in the pixel; *B*: baseline distance; *f*: focal length. $x_{wi}$, $y_{wi}$, $z_{wi}$: Object's 3D world coordinate of the *i* th frame. *n*: maximum number of frames of the video. (B) Processing of 3D coordinate estimation using 'triangulate' function.

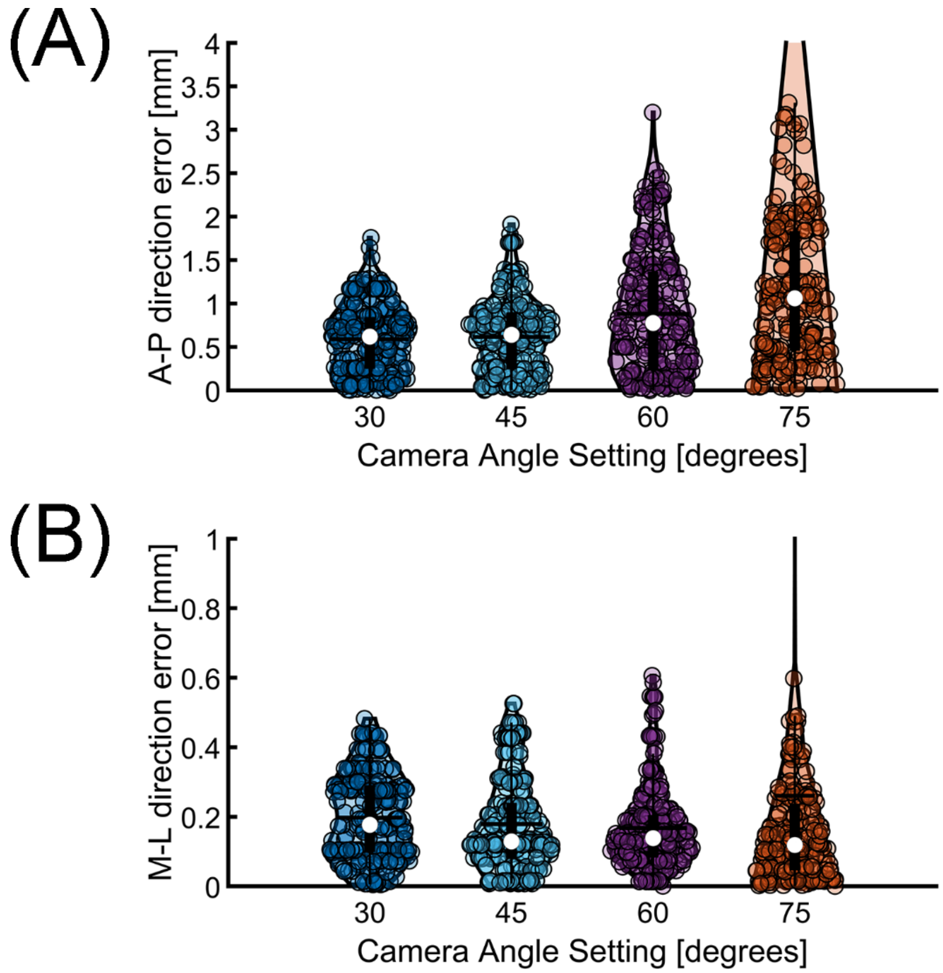


**Supplementary Figure 5. Camera angle validation experiment results.** (A) Violin plots for distance estimation errors in anterior-posterior (A-P) direction. Camera angle settings indicate the angle between the camera plane and the table. Extreme estimation error values at a 75-degree angle are not shown to represent data from other angles better. (B) Violin plots for distance estimation errors in mediolateral (M-L) direction. Extreme estimation error values at a 75-degree angle are not shown to represent data from other angles better.

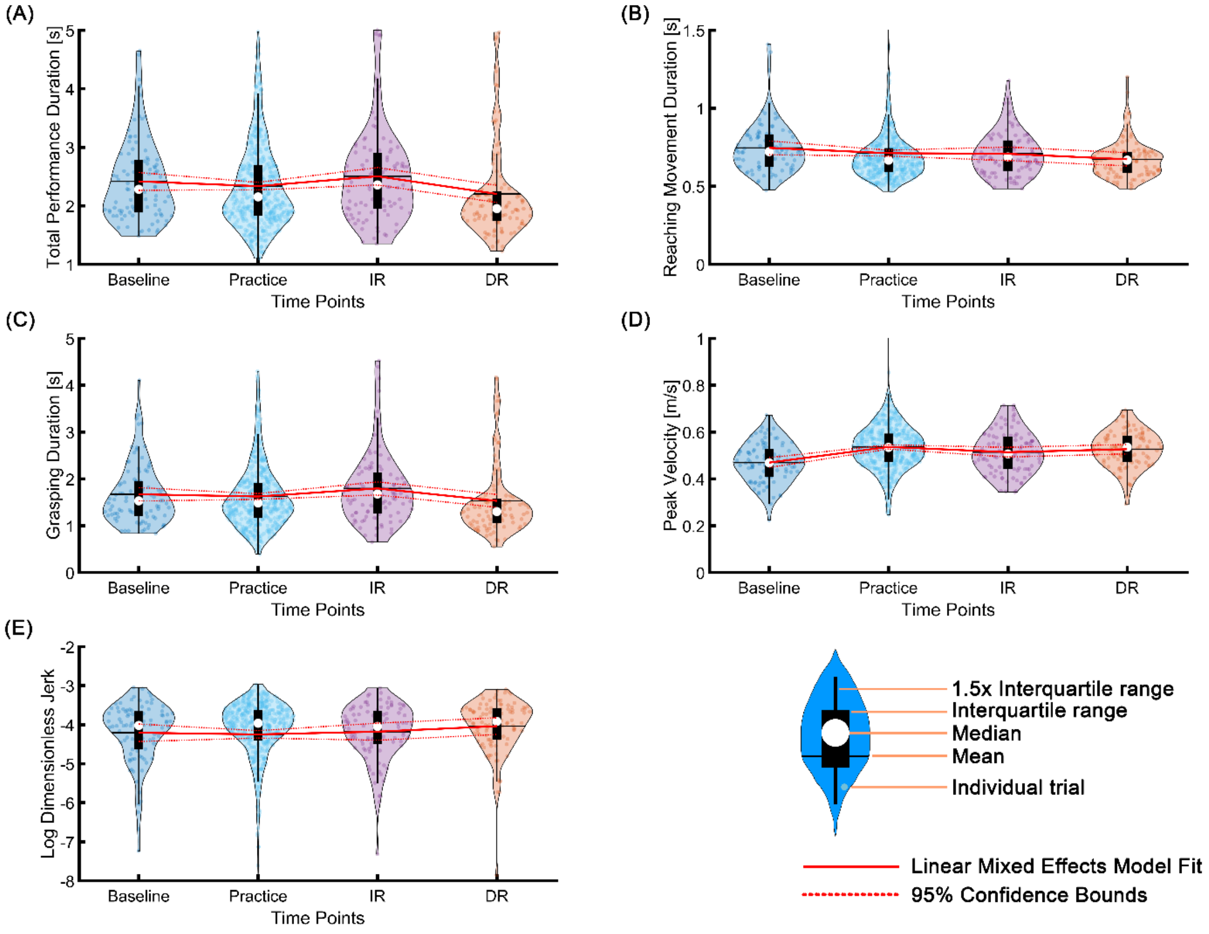


**Supplementary Figure 5.** Experiment 3 results. (A) Total performance duration (PD). Scatter plots indicate individual trials. A solid red line represents the linear mixed effects model fitting, and red dotted lines indicate the 95% confidence interval of the model fitting. (B) Object grasping duration (GD). (C) Object movement duration (MD). (D) Peak tangential velocity amplitude (PV). (E) Log dimensionless jerk (LDJ).

**Supplementary Table 2. Camera Video Capture Settings.**

| Parameters | Experiment 1 | Experiment 2 | Experiment 3 |
|---|---|---|---|
| Resolution | 2.7K | 2.7K | 2.7K |
| Frame Rate (fps) | 24 | 60, 120 | 120 |
| Lens | Linear | Linear | Linear |
| Protune color | Flat | Flat | Flat |
| Bit rate | High | High | High |
| White balance | 5,000K | 5,000K | 5,000K |
| ISO minimum | 100 | 100 | 100 |
| ISO maximum | 1,600 | 1,600 | 1,600 |
| EV compensation | Not set | Not set | Not set |
| Sharpness | Medium | Medium | Medium |
| Lock Shutter (Shutter Speed) | Auto | 45° (1/960 s) | 45° (1/960 s) |
| The angle between the camera sensor plane and target plane | 0° | 0° | 30° |
| Stereo Camera Baseline Distance | 10, 12.5, 15, 17.5, and 20 cm | 15 cm | 15 cm |
| Distance between the camera 1 and the target object | 30 – 100 cm | ~ 50 cm | ~ 40 – 60 cm |

**Supplementary Table 3. Pendulum Simulation Parameters.**

| Pendulum Parameters | Setting |
|---|---|
| Bob mass | 50 kg |
| Rod length | 2 m |
| Gravitational acceleration | 9.81 $m/s^2$ |
| Damping coefficient | 0.25 |
| Simulation time | 10 seconds |
| Simulation time step | 1/120 seconds |
| Initial angular position | $\pi/2$ |

**Supplementary Table 4. Demographics Summary of Participants in Experiment 3.**

| Variables | Average ± Standard Deviation [Min – Max] Or Count |
|---|---|
| Age | 26 ± 2 [22 – 28] |
| Sex | Male: 3<br>Female: 8 |
| Race | White: 5<br>Asian: 5<br>Hispanic: 1 |
| Hand Dominance | Right: 11<br>Left: 0 |
| Previous Chopstick Experience | Yes: 8<br>No: 3 |
| Dominant Hand Chopstick Operation Self-Efficacy* | 69.36 ± 31.53 [11 – 100] |
| Non-dominant Hand Chopstick Operation Self-Efficacy | 29.18 ± 21.21 [0 – 66] |

* The chopstick operation self-efficacy was measured using a questionnaire asking how confident is the participant to pick up a plastic cube (1 cm on edge) using a pair of chopsticks with each hand.

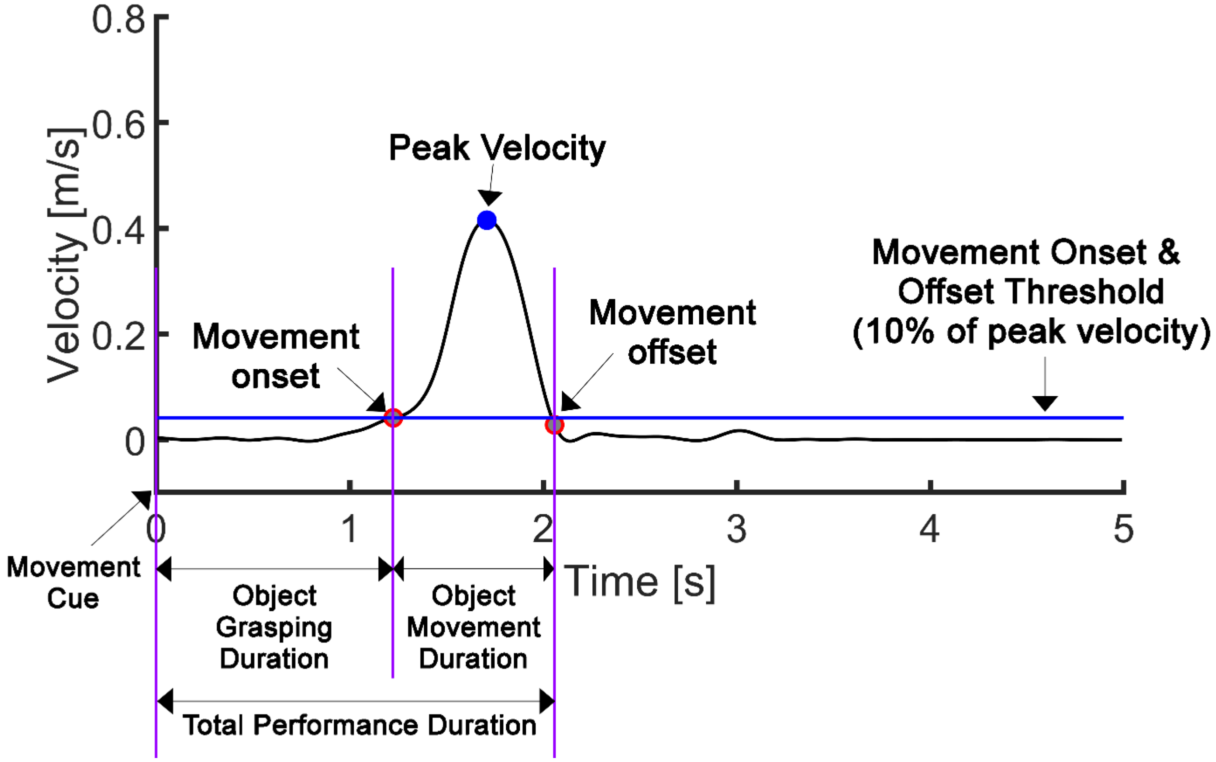


Supplement Figure 6. Kinematic Analysis in Experiment 3. The resultant from the 3-D object position data was utilized to determine the object's tangential velocity for each chopstick trial (first derivative of the resultant). The peak velocity was then determined, and the movement onset/offset threshold was determined to be 10% of the peak velocity. Temporal kinematic variables, such as object grasping duration, object movement duration, and total performance duration, were estimated using the movement onset and offset.

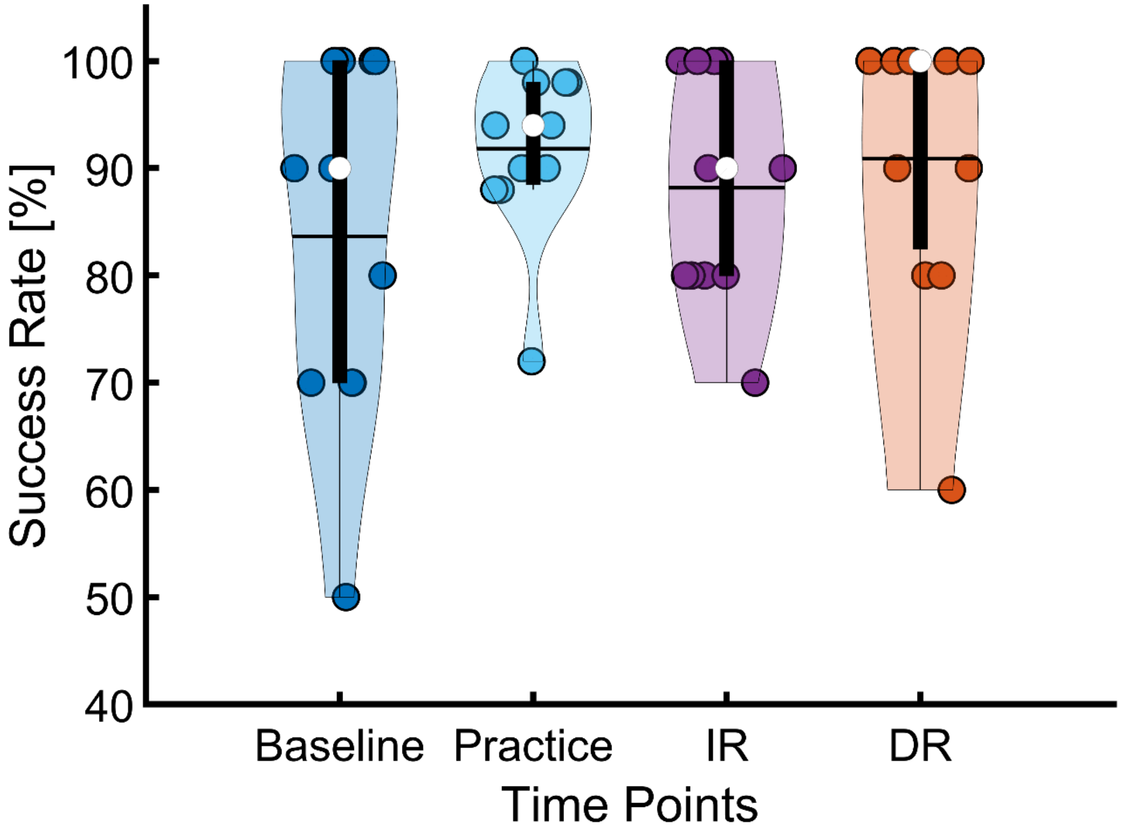


Supplement Figure 7. Success rate of chopstick motor skill test at different time points. Individuals are represented using scatter plots. The white dots represent the median success rate at each time point. The black horizontal line denotes the mean success rate at each time point. IR: immediate retention; DR: delayed retention.
